# Detection of human papillomavirus distinguishes second primary tumors from lung metastases in patients with squamous cell carcinoma of the cervix

**DOI:** 10.1111/1759-7714.13544

**Published:** 2020-07-03

**Authors:** Suxia Lin, Xinke Zhang, Xiaoxuan Li, Changfei Qin, Lihong Zhang, Jiabin Lu, Qunxi Chen, Jietian Jin, Taoli Wang, Fang Wang, Shengbing Zang

**Affiliations:** ^1^ State Key Laboratory of Oncology in South China, Collaborative Innovation Center for Cancer Medicine Sun Yat‐sen University Cancer Center Guangzhou China; ^2^ Department of Pathology Sun Yat‐sen University Cancer Center Guangzhou China; ^3^ Guangdong Esophageal Cancer Institute Guangzhou China; ^4^ Department of Pathology The Seventh Affiliated Hospital of Sun Yat‐sen University Shenzhen China; ^5^ Department of Pathology Zhuzhou Central Hospital Zhuzhou China; ^6^ Department of Molecular Diagnostics Sun Yat‐sen University Cancer Center Guangzhou China

**Keywords:** Human papillomavirus, lung metastasis, RNAscope assay, second primary tumor, squamous cell carcinoma

## Abstract

**Background:**

In patients with squamous cell carcinoma of the cervix (SCCC), a squamous cell carcinoma (SqCC) in the lung represents either a second primary tumor or metastasis. This distinction between second primary tumors and lung metastases in patients with SCCC significantly influences patient prognosis and therapy. Here, we aimed to differentiate second primary tumors from lung metastases in patients with SCCC by exploring the HPV status in SqCCs involving the lung within a large cohort.

**Methods:**

P16 expression was assessed using immunohistochemistry on tissue microarrays including a total of 415 primary lung SqCCs and 21 lung SqCCs with prior SCCC. Following this, we performed HPV DNA typing and the sensitive RNAscope in situ method to screen all the cases for HPV E6/E7 expression, which is a more reliable indicator of transcriptively active HPV in tumor cells.

**Results:**

The p16 positive expression rate was 13.7% (57/415) in primary lung SqCCs, but HPV DNA was not detected in any of the 57 primary lung SqCC cases that positively expressed p16. In contrast, HPV DNA was detected in all cases (21/21) with prior SCCC. Consistently, all 21 lung SqCCs with prior SCCC (21/21) showed extensive HPV16 E6/E7 expression. In striking contrast, none of the primary lung SqCCs (0/415) had a detectable RNAscope signal.

**Conclusions:**

HPV does not seem to play a role in the development of primary lung SqCCs. HPV detection may be helpful in distinguishing second primary tumors from lung metastases in patients with SCCC.

## Graphical abstract

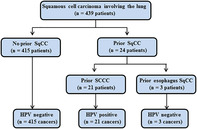

## Introduction

Squamous cell carcinoma of the cervix (SCCC) accounts for 70%–80% of all cervical cancer cases, which is the fourth most frequent cancer type in women worldwide.^1^ Lung metastases is frequently observed in patients with SCCC and is an indicator of the late clinical stage with no options for curative intervention, whereas a second primary lung squamous cell carcinoma (SqCC) in patients with SCCC may be of a lower stage and resectable.^2^, ^3^ Therefore, the ability to distinguish between a second primary tumor and metastasis is a critical step in guiding clinical therapy. However, it is very challenging to distinguish second primary tumors from lung metastases in patients with SCCC, since SqCCs of various sites have similar morphologic appearance to dyskeratotic cells and squamous pearls. Novel strategies based on molecular genetic fingerprinting such as microsatellite alterations and gene mutations have been introduced to clarify the distinction between a second primary tumor and lung metastasis. However, these novel strategies have been limited in routine clinical practice owing to their cost, technical feasibility, and the inherent instability of the genetic profile of a tumor over time. To date, no optimally convenient and reliable approaches are available.

The overexpression of p16 is strongly associated with the infection of high‐risk human papilloma virus (hrHPV), and has been used as a surrogate marker for predicting the HPV status in the head and neck SqCCs^4^, ^5^ and SCCCs.^6^ However, the correlation between p16 and HPV infection is not well established in primary lung SqCC. Previous studies have implicated the overexpression of p16 in some patients with primary lung SqCC,^7^, ^8^, ^9^ whereas the detection rates of HPV have been reported to widely range from 0% to 78% in primary lung SqCC.^10^, ^11^ The significantly variable ranges of HPV infection rate in lung SqCCs is due, in part, to HPV detection methods.^8^, ^12^ The most commonly used methods to detect HPV DNA include polymerase chain reaction (PCR) and in situ hybridization. However, the presence of viral DNA does not necessarily confirm a carcinogenic role for the cause of the lesion.^8^, ^12^ To this purpose, demonstrating the presence of transcriptively active HPV E6 and E7 viral oncogenes is more informative. In our study, we combined immunohistochemistry for p16, HPV DNA typing, and an in situ mRNA staining technique able to detect HPV E6 and E7 gene expression, in order to clarify the HPV infection status in the lung SqCC cases within a large cohort.

The objective of this study was to explore the correlation between p16 and the HPV status in primary lung SqCCs with a large cohort, and to differentiate second primary tumors from lung metastases in patients with SCCC.

## Methods

### Case details

The study was approved by the Sun Yat‐sen University Cancer Center Medical Ethics Committee. It was conducted using data from a lung SqCC database composed of 415 consecutive primary lung SqCC cases that underwent surgical treatment between January 2011 and December 2015 at the Sun Yat‐sen University Cancer Center. These cases were clinically considered primary lung cancers without any prior SqCCs in other sites as well as HPV infection history.

Histological diagnosis was performed according to the 2015 World Health Organization classification of lung tumors.^13^ All primary lung SqCC cases included in this analysis were restaged according to the eighth edition of the American Joint Committee on Cancer (AJCC) lung cancer stage classification.^14^ Clinical information was correlated with the study findings.

We also collected 21 lung SqCCs with prior SCCC and three lung SqCCs with prior esophageal SqCC between January 2011 and December 2015 at the Sun Yat‐sen University Cancer Center.

### Tissue microarray construction

Tissue microarray (TMA) was constructed as previously described.^15^ Briefly, the most representative area was marked on a conventional paraffin tissue block from each case and was then transferred to the recipient block using a TMA machine (Minicore Excilone, Minicore, UK). A total of 415 formalin‐fixed, paraffin‐embedded (FFPE) primary lung SqCCs, 21 lung SqCCs with prior SCCC and three lung SqCCs with a prior esophageal SqCC were prepared for TMA construction. Three punch cores of 1.0 mm of each case were transferred to the TMA block, yielding a total of 11 TMA blocks. TMA serial sectioning was carried out in 4 μm using a microtome.

### Immunohistochemistry (IHC)

Both the TMA and the corresponding FFPE tissue slides were deparaffinized in xylene, rehydrated through graded alcohol, immersed in 3% hydrogen peroxide for 10 minutes to block endogenous peroxidase activity, and the antigen retrieved by pressure cooking for three minutes in citrate buffer (pH = 6). For blocking nonspecific binding, the slides were preincubated with 10% normal goat serum at room temperature for 20 minutes. Subsequently, the slides were incubated with mouse monoclonal antibody against human p16 (clone INK4a/E6H4, 1:200 dilution, Ventana, AZ, USA), overnight at 4°C in a moist chamber. The slides were then sequentially incubated with a secondary antibody (Envision, Dako, Denmark) for 30 minutes in the incubator at 37°C, and stained with DAB (3,3‐diaminobenzidine). Finally, the sections were counterstained with Mayer's hematoxylin, dehydrated and mounted. A negative control was obtained by replacing the primary antibody with a normal mouse IgG antibody. The positive expression of p16 was defined as equal to or more than 70% of neoplastic cells with both nuclear and cytoplasmic staining.^16^ Stained slides were reviewed independently by two pathologists (XL and SZ), and a consensus staining result was reached for each case.

### DNA extraction

FFPE tumor tissue sections were reviewed through hematoxylin‐eosin (HE) staining, and regions containing more than 70% tumor cells on unstained sections were selected for macrodissection by an expert pathologist (XZ). Genomic DNA was extracted using the QIAamp DNA FFPE Tissue kit (Qiagen, Hilden, Germany). DNA was extracted according to the manufacturer's recommendations. Briefly, four 10 μm FFPE sections were submitted to deparaffinization with 100% xylene, followed by washes of ethanol (100%, 90%, and 50%) and incubation with DNA extraction buffers overnight. All DNA samples were quantified by a NanoDrop 2000 (Thermo Scientific, Massachusetts, USA).

### HPV DNA detection and typing

Detection of HPV was performed with a HPV DNA detection kit (PCR‐ fluorescence probing, Tragene Medical, China). For PCR, detection range were HPV type 6, 11, 18, 26, 31, 33, 35, 39, 45, 5, 52, 53, 56, 58, 59, 66, 68, 73, 81, 82. DNA was amplified in a total volume of 5 ng/μl of reaction mixture. PCR was performed using the following protocol: 95°C for 30 seconds, 95°C for 5 seconds and 65°C for 20 seconds, for 10 cycles; 95°C for 5 seconds and 32°C for 20 seconds for 30 cycles. The detection was performed using the 7500 real‐time quantitative PCR system (Applied Biosystems, Foster City, CA, USA). Fluorescence detection dyes included FAM, VIC, ROX, and CY5. Interpretation criteria: internal reference gene amplification with Ct ≤25. The amplification curve of the test sample showed a typical S‐shape, and the Ct value was ≤28, which was positive. Appropriate positive and negative controls were included in the detection according to the manufacturer's instructions. The amplification of the positive control gene indicated the integrity of the DNA for subsequent HPV analysis.

### In situ detection of HPV transcription using RNAscope assay

To test the high‐risk HPV (HR‐HPV) specific transcripts, we used RNAscope 2.5 assay HPV kit and the HPV‐HR18 probe cocktail (Advanced Cell Diagnostics Inc., Hayward, CA, USA) according to the manufacturer's instructions. Briefly, after deparaffinization and pretreatment with heat and protease, 4 μm TMA slides were incubated with the HPV‐HR18 probe cocktail which targeted the E6 and E7 transcripts from 18 HR‐HPV genotypes (HPV16, 18, 26, 31, 33, 35, 39, 45, 51, 52, 53, 56, 58, 59, 66, 68, 73, and 82). Then, after fully washing, slides were counterstained with HE and recorded as either positive or negative. A positive control probe, Pol2A, detecting the human housekeeping gene transcript was used for all the cases to confirm the integrity of the RNA. DapB probes which can only recognize bacterial transcripts were used as negative control. Paraffin sections of HeLa cells were used as an experimental systemic control.

Stained slides were reviewed independently by two pathologists (XL and SZ), and a consensus staining result was reached for each case. RNAscope result was considered positive when brown punctate staining in the cytoplasm and the nucleus of tumor cells occurred.^4^

### Statistical analysis

Statistical analyses were performed using SPSS software, version 16.0 (SPSS, Chicago, USA). The Kaplan‐Meier method was used to assess the association between clinicopathological variables and overall survival (OS) of lung SqCC patients in a univariate analysis. A two‐tailed *P*‐value less than 0.05 was considered statistically significant in all cases.

## Results

### Patient characteristics of SqCC involving the lung

Clinical characteristics of the patients with primary lung SqCCs are summarized in Table 1. A total of 415 patients with primary lung SqCC were included in this study. There were 401 men and 14 women aged from 35 to 79 years (median, 61 years). Cell differentiation in 96.9% (402/415) of the patients was moderate to poor grade and 47.7% (198/415) tumors were located in the right lung. Among the patients, 29.9% (124/415) of the diagnoses were estimated to be at III to IV stage. There were no significant differences with regard to the distribution of gender, age, cell differentiation, tumor location, and tumor size between the different p16 expression categories.

**Table 1:** Relationship between p16 expression and clinicopathologic parameters of primary lung squamous cell carcinoma (SqCC) patients

	P16 positive	P16 negative	
Variable	Patient No. (%)	Patient No. (%)	*P*‐value
Gender			0.395
Male	54 (13.5%)	347 (86.5%)	
Female	3 (21.4)	11 (78.6%)	
Age (years)			0.724
≤ 60	25(13.1%)	166 (86.9%)	
> 60	32(14.3%)	192 (85.7%)	
Cell differentiation			0.525
Well	1 (7.7%)	12 (92.3%)	
Moderate	34 (12.8%)	232 (87.2%)	
Poor	22 (16.2%)	114 (83.8%)	
Tumor location			0.956
Right lung	27 (13.6%)	171 (86.4%)	
Left lung	30 (13.8%)	187 (86.2%)	
Tumor size (cm)			0.599
0–4.6	34 (14.7%)	201 (85.5%)	
> 4.7	23 (12.8%)	157 (87.2%)	
Pathological stage			0.144
I	16 (12.5%)	112 (87.5%)	
II	18 (11.0%)	145 (89.0%)	
III	23 (19.5%)	95 (80.5%)	
IV	0 (0.0%)	6 (100.0%)	

A total of 21 patients were identified who had a primary SCCC and went on to develop SqCC in their lungs. The median age at diagnosis was 52 (range 42–64) and 19.0% (4/21) of the patients were older than 60 years. The time intervals from treatment of the SCCC to detection of a SqCC in the lungs ranged from 15 to 123 months (median, 26 months). In 15 cases, the lung tumors presented as a solitary mass, whereas in six cases there were multiple lung nodules. Three patients, aged 62, 66, and 68 years, were identified as having a primary esophageal SqCC and went on to develop SqCC in their lungs. The time interval from treatment of the esophagus to detection of a SqCC in the lung was 21, 59, and 118 months.

### p16 expression in SqCCs involving the lung

Clinicopathological results including p16 expression from IHC are detailed in Table 1. p16 positive expression rate was 13.7% (57/415) in the TMAs of primary lung SqCCs, which was consistent with the results of using the conventional paraffin tissue block from each case. The 57 patients with p16 expression were aged 35.0 to 79.0 years old (one case was 35.0 years, 56 cases ranged from 46.0 to 79.0 years), and the mean was 61.0 years. The relationship between p16 expression and selected clinicopathologic parameters is shown in Table 1. P16 expression did not vary significantly with gender, age, cell differentiation, tumor location, tumor size, or pathological stage.

**Figure 1:**
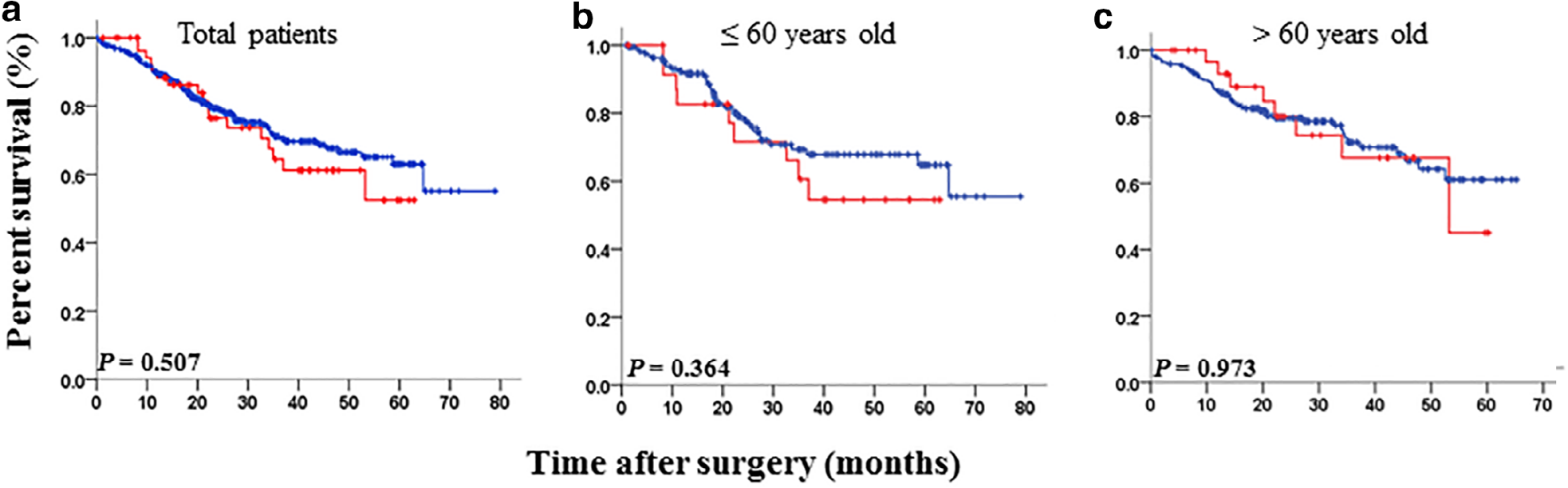
Kaplan‐Meier analysis of overall survival for primary lung squamous cell carcinoma (SqCC) patients with p16 expression. (**a**) Overall survival curves for two categories of patients according to p16 expression in the 415 patients with primary lung SqCC (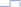) p16 negative (*n* = 358), (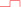) p16 positive (*n* = 57); (**b**) Overall survival curves for two categories of patients according to p16 expression in the 191 patients with primary lung SqCC aged less than or equal to 60 years old (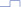) p16 negative (*n* = 166), (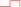) p16 positive (*n* = 25); (**c**) Overall survival curves for two categories of patients according to p16 expression in the 224 patients with primary lung SqCC aged more than 60 years old (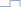) p16 negative (*n* = 192), (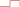) p16 positive (*n* = 32).

The median follow‐up time was 49.4 months，ranging from 1.4 to 191.1 months for the entire cohort of patients. From the results of the Kaplan‐Meier statistical analysis, the survival rates of the p16 positive expression group and the p16 negative expression group both declined progressively and there was no significant difference in the overall survival of the primary lung SqCC patients between these two groups (*P* = 0.507, Fig 1(a)). In addition, there was no significant difference in the overall survival of the primary lung SqCC patients less than 60 years old (*P* = 0.364, Fig 1(b)) or more than 60 years old (*P* = 0.973, Fig 1(c)) between the p16 positive expression as well as the p16 negative expression groups. Thus, the expression of p16 is not associated with multiple malignant characteristics of primary lung SqCCs, and p16 does not seem to play a critical role in the development of primary lung SqCC.

### P16 expression and HPV status in SqCCs involving the lung

**Figure 2:**
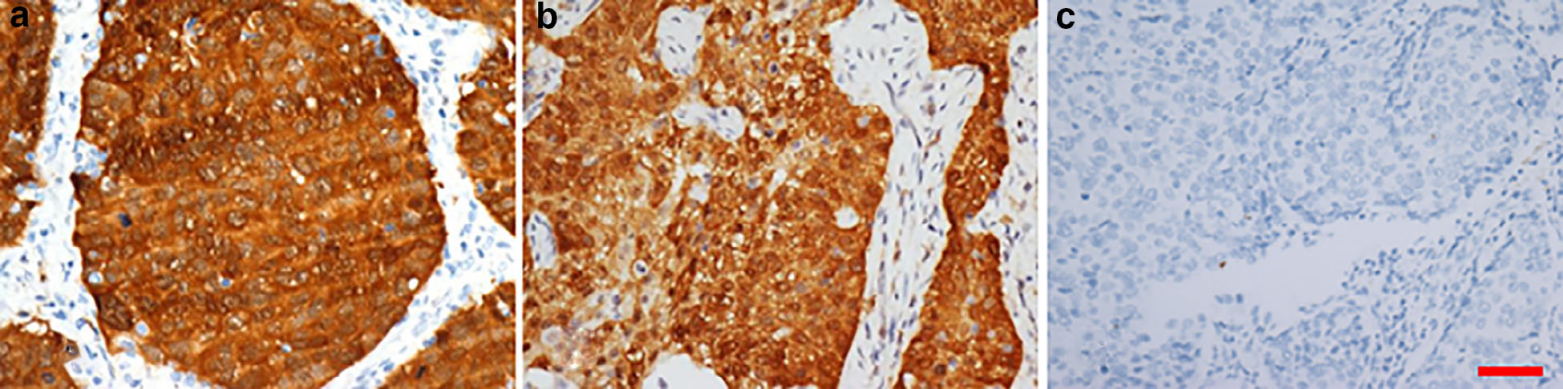
P16 expression in squamous cell carcinoma (SqCC) involving the lung. (**a**) P16 expression in primary lung SqCC. (**b**) P16 expression in lung SqCC with a prior SCCC. (**c**) A negative control using a normal mouse IgG instead of p16 antibody. EliVision Plus two‐step immunohistochemical technique with 3–3′ diaminobenzidine (DAB) staining was used; Original magnification: × 400; scale bar: 10 μm.

To determine the association of p16 expression with HPV infection in SqCCs involving the lung, we performed HPV DNA typing in 57 primary lung SqCCs in which p16 was positively expressed (Fig 2(a)), 21 lung SqCCs in which p16 was positively expressed (Fig 2(b)) with prior SCCC, and three lung SqCCs with prior esophageal SqCC. The results of the HPV analysis are summarized in Table 2. A total of 21 patients were identified who had a primary SCCC and went on to develop lung SqCC. Three patients developed lung SqCC after they had primary esophageal SqCC. All 21 cases with prior SCCC were HPV DNA‐positive including 16 cases with HPV type 16, three cases with type 18, and two cases with types 73/35/81 (without specific type) and 16 plus 31, respectively. The HPV type in the lung SqCC and the corresponding original cervical lesion was the same. None of the lung SqCCs (0/3) with prior esophageal SqCC were HPV DNA‐positive; they were also HPV DNA‐negative in the corresponding original esophageal lesions. Notably, HPV DNA was not detected in any of the 57 cases of primary lung SqCCs with p16 positive expression. Clearly, p16 expression is not associated with the presence of HPV in primary lung SqCC and HPV analysis could help distinguish lung metastases of cervical but not esophageal origin from primary lung SqCC.

**Table 2:** P16 expression and HPV status of lung SqCC

Lung SqCC	p16 IHC (%)	HPV DNA
No prior SCCC	57/415 (13.7)	0/57† (0)
Prior SCCC	21/21 (100%)	21/21(100%)
Prior esophagus SqCC	1/3 (25%)	0/3 (0)

† 57 primary lung SqCCs with p16‐ positive expression.

SqCC, squamous cell carcinoma; SCCC, squamous cell carcinoma of cervix.

### In situ detection of HPV transcription in SqCCs involving the lung

**Figure 3:**
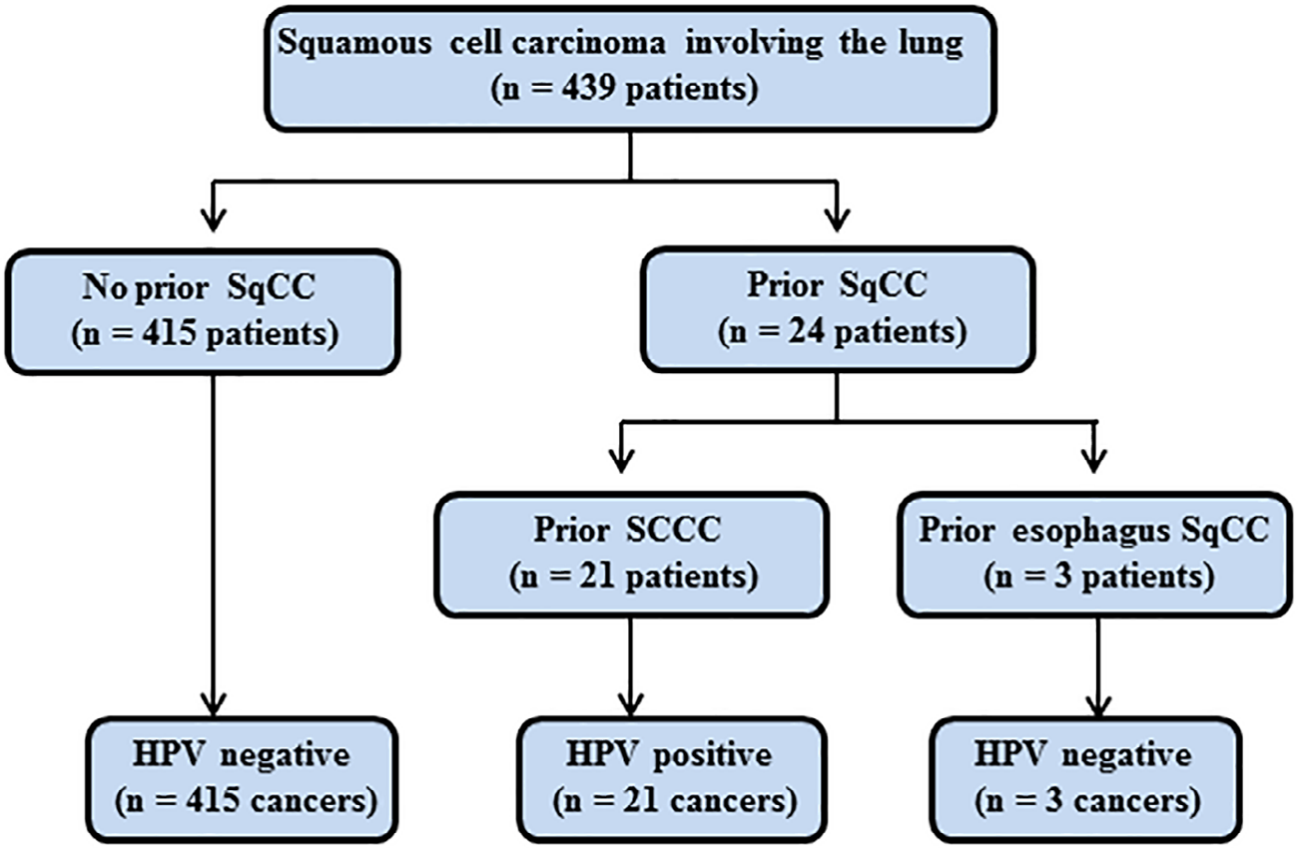
In situ detection of HPV transcription in squamous cell carcinoma (SqCC) involving the lung of patients with or without a prior SqCC of cervix or esophagus. HPV detection is strictly limited to those patients with a prior SCCC. SqCC, squamous cell carcinoma; SCCC, squamous cell carcinoma of cervix.

**Figure 4:**
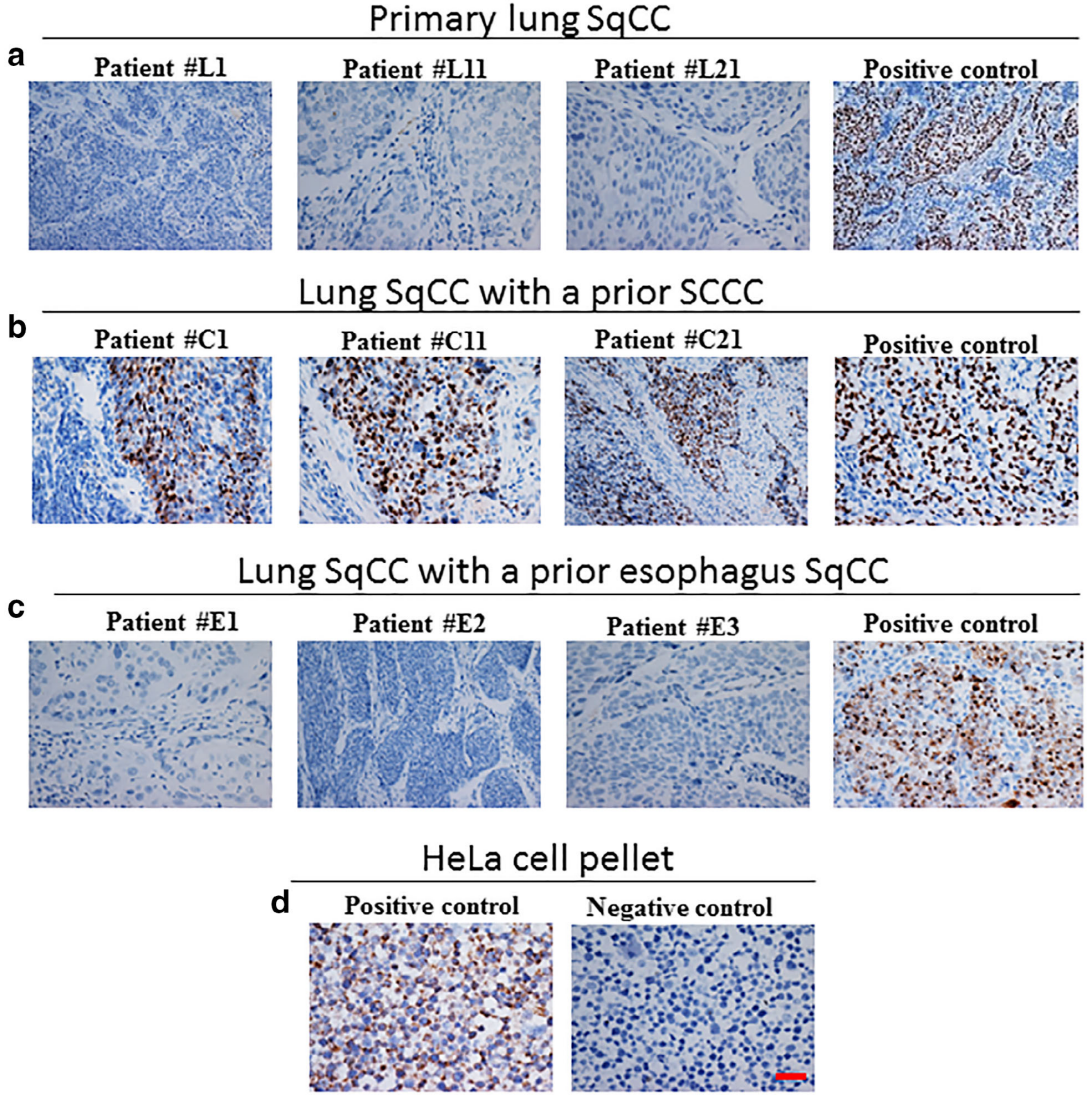
In situ detection of HPV transcription in squamous cell carcinoma (SqCC) involving the lung. (**a**) HPV E6/E7 expression was not detectable in the primary lung SqCCs. (**b**) HPV E6/E7 expression was detectable in the lung SqCCs with a prior SCCC. (**c**) HPV E6/E7 expression was not detectable in the lung SqCCs with a prior esophagus SqCC. (**d**) Paraffin sections of HeLa cells were used as an experimental systemic control. Positive control was set in each group.

Since the presence of viral DNA does not necessarily confirm a carcinogenic role in the development of SqCC, we employed the sensitive RNAscope in situ method to screen all the cases for HPV gene expression. The integrity of RNA of all the cases was identified by using a positive control probe, Pol2A housekeeping gene. All the sections showed evidence of housekeeping gene expression in the tumor cells to indicate the intact RNA and thus the extent of the HPV E6/E7 gene expression was subsequently assessed. DapB‐negative control probes performed in all cases were negative. As shown in Fig 3, none of the primary lung SqCC (0/415) had a detectable RNAscope signal (Fig 4(a)). In striking contrast, the entire 21 cases of lung SqCCs with prior SCCC (21/21) showed extensive HPV E6/E7 expression (Fig 4(b)) which was also found to be positive in the corresponding original SCCCs (Fig S1a). As expected, none of the lung SqCCs (0/3) with prior esophageal SqCC showed HPV E6/E7 expression (Fig 4(c)) which was also found to be negative in the corresponding original esophageal lesions (Fig S1b). The RNAscope results are consistent with the HPV DNA detection on the lung primary and metastatic squamous cell carcinomas. In total, our data indicated that detection of HPV by the RNAscope assay distinguished lung metastases of cervical origin from primary lung squamous cell carcinomas. However, since HPV E6/E7 expression was not detected in both lung metastases of esophageal origin and primary lung squamous cell carcinoma, it is not useful for a differential diagnosis.

## Discussion

In patients with SCCC, a SqCC in the lungs represents either a second primary tumor or metastasis. This distinction significantly influences patient prognosis and could guide treatment strategies, but differentiation between second primary tumors from lung metastases in patients with SCCC can, at times, be very challenging because both of these tumors can appear morphologically identical. Our results showed that the p16 positive expression rate was approximately 13.7% (57/415) in patients with primary lung SqCC. Therefore, the application of the p16 protein as an indicator to differentiate second primary tumors from lung metastases in patients with SCCC is not feasible because both tumors can be p16‐positive. Given that HPV is an established causative agent for SCCC, we urgently need to explore the HPV status in lung SqCCs in order to distinguish the second primary tumors from lung metastases in patients with SCCC.

There was no HPV infection in the 57 primary lung SqCC cases with p16‐positive expression in our study, using PCR for HPV DNA, which is consistent with previous studies that have shown a lack of HPV infection in the primary lung SqCCs.^4^, ^8^ Chang *et al*. reported that they did not detect HPV infection by DNA‐ISH or RNA‐ISH in 96 cases of primary SqCC, despite positive p16 expression in a portion of the SqCCs.^8^ Bishop *et al*.^4^ investigated 220 cases of SqCCs involving the lung with HPV DNA by ISH. Of these, 166 had no known history of head and neck primary cancer, and no HPV was detected in their lung SqCCs. In the remaining 54 patients with a known history of head and neck primary SqCC, HPV was found in 11 of the cases. Of the 11 cases, 10 were HPV positive in both lung and head and neck SqCC (data unknown for one case); the latter result was not surprising due to partial head and neck primary SqCCs harboring the HPV infection. Doxtader *et al*.^17^ also did not detect HPV, using DNA‐ISH, in 20 primary lung SqCCs. Although the above mentioned studies indicate the absence of any HPV DNA type in primary lung SqCC, other research has reported frequencies of HPV infection in lung cancer, which varies worldwide,^11^, ^18^ ranging from 7.3% to 41.2%, as well as 36.2% in Asia‐Pacific patients with lung SCC. Klein *et al*.^18^ highlighted that the wide‐ranging prevalence rates have not been associated with any factors, including the country, continent, time studied, technique, and sample type. Weichart *et al*.^2^ conducted an HPV analysis using both chromogenic in situ hybridization (CISH) and a PCR array to investigate the existence of HPV positive lung SqCC. Therefore, the detection of HPV DNA may not effectively identify the metastases entity, although our study reported no HPV infections in the 57 lung primary SqCC cases, using PCR for HPV DNA. Based on the above evidence, it is practically impossible to distinguish between SqCCs of different primary sites by using HPV DNA detection.

Therefore, the effect of HPV on the oncogenesis of lung SqCC is unclear. However, PCR detection for E6/E7 mRNA transcripts requires good‐quality mRNA, which is especially challenging in FFPE tissues. Conversely, the RNAscope, a new method is not limited by mRNA quality which can directly visualize E6/E7mRNA transcripts in tumor cells on FFPE histologic sections by ISH. Our studies verified that none of the 415 lung primary SqCC had a detectable RNAscope signal, and that all 21 cases of lung metastatic SCCC showed extensive HPV16 E6/E7 expression. Consistent with our findings, Van Boerdonk *et al*.^19^ used RT‐PCR to detect the presence of HPV‐E7 mRNA in 85 primary lung SqCC, but failed to detect any HPV infections and Chang *et al*. found that HPV DNA‐ISH and RNA‐ISH were both negative in nine cases of lung SqCC.^8^ More importantly, Coissard *et al*.^20^ explored 218 fresh frozen lung tumors for the presence of HPV with the Roche line blot assay and the expression of mRNAs encoding E6 oncoprotein in HPV positive tumors. Only four samples were positive for HPV detection; one poorly differentiated squamous cell carcinoma and three large cell carcinomas. E6 mRNA was undetectable in the four samples. These data confirm the low prevalence of HPV in lung carcinomas and are not favorable for concluding that there is a carcinogenic role for HPV in these carcinomas. Therefore, we were able to identify the transcriptively active HPV‐infected cases by RNAscope assay in clinical practice.

In addition, we did not detect HPV DNA and RNA in any of the primary lung SqCCs with or without p16 positive expression, indicating that p16 expression is not related to HPV infection. This also suggests that HPV does not play a central role in lung carcinogenesis, which is consistent with a previous study.^21^ This led us to speculate that p16 positive expression of partial patients with SqCC could be associated with other mechanisms, such as p16 promoter methylation, which is a general epigenetic alteration that has been previously shown to be involved in NSCLC.^22^ Another study reported decreased promoter hypermethylation of p16 in patients less than 40 to 50 years of age when compared with older patients,^23^ indicating p16 overexpression is involved in patients younger than 50 years old but that there is a lack of p16 promoter hypermethylation. However, this result is not consistent with our findings as there was widespread existence of p16 positive expression in patients more than 50 years old; therefore, we suggest that methylation‐independent mechanisms control the gene expression at the p16 locus, including transcription factors and long noncoding RNA.^24^, ^25^ We found that p16 expression status was not associated with the prognosis of all patients with lung SqCC, which is consistent with the findings in a previous study by Zhou *et al* ^7^ These results indicate the significance of the p16 protein; however, its involvement in lung SqCC remains to be further analyzed. Moreover, the definitive role of HPV in lung carcinogenesis needs to be determined.

In summary, this study demonstrated that p16 protein is not a surrogate marker for HPV infection in lung SqCC among East Asian SqCC patients. We also determined that HPV does not play a critical role in lung carcinogenesis, and that HPV‐positive cancers mostly occur in patients with a history of HPV‐related cancers located at other sites. Most importantly, detection of HPV by RNAscope assay could distinguish more accurately between lung metastases of cervical origin and primary lung SqCC.

## Disclosure

No authors report any conflict of interest.

## Supporting information


**Figure S1** In situ detection of HPV Transcription in SCCC and esophageal SqCC. (**a**) HPV E6/E7 expression was detectable in the SCCC. (**b**) HPV E6/E7 expression was not detectable in the esophageal.
