# Supplementary figures and images for: Detection of human papillomavirus distinguishes second primary tumors from lung metastases in patients with squamous cell carcinoma of the cervix
Source: Thorac Cancer. 2020 Jul 3;11(8):2297–305. doi: 10.1111/1759-7714.13544 (PMC7396378; doi:10.1111/1759-7714.13544)

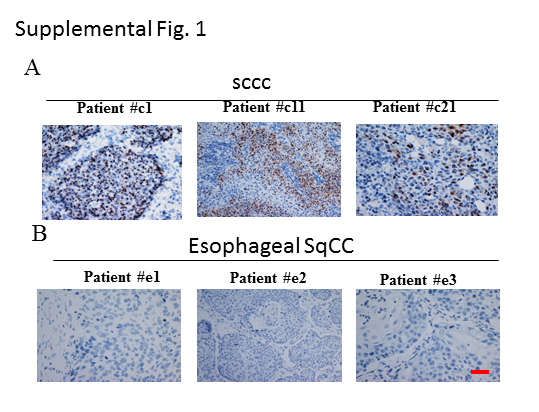

Supplement: Supplementary file 1 — Figure S1 In situ detection of HPV Transcription in SCCC and esophageal SqCC. (a) HPV E6/E7 expression was detectable in the SCCC. (b) HPV E6/E7 expression was not detectable in the esophageal. [file TCA-11-2297-s001.tif]
